# Prognostic Value of Hyperechoic Echo Halo in cN0 Papillary Thyroid Microcarcinoma and Its Correlation with Age and Gender

**DOI:** 10.1155/2020/6479582

**Published:** 2020-02-27

**Authors:** Xiaojuan Zheng, Yunwen Jiang, Chenyin Zhao, Minxia Peng, Liyong Qian

**Affiliations:** ^1^Department of Ultrasonography, Zhoushan Hospital of Wenzhou Medical University, Zhoushan, Zhejiang Province, China; ^2^Department of Ultrasonography, Tongde Hospital of Zhejiang Province, Hangzhou, Zhejiang Province, China; ^3^Department of Thyroid Surgery, Zhoushan Hospital of Wenzhou Medical University, Zhoushan, Zhejiang Province, China; ^4^Department of Pathology, Zhoushan Hospital of Wenzhou Medical University, Zhoushan, Zhejiang Province, China

## Abstract

*Purpose*. To investigate the pathology and prognostic value of hyperechoic echo halo in cN0 papillary thyroid microcarcinoma (PTMC) and the relationship between age, gender, and the formation of abnormal hyperechoic echo halo and cervical lymph node metastasis. Data of 97 patients who underwent surgical treatment for the first time for single PTMC between April 2016 and March 2017 were analyzed retrospectively. The boundary status of the PTMC was determined preoperatively. Grayscale values of the nodular center, hyperechoic echo halo, and normal thyroid tissue were acquired with Adobe Photoshop CS6 software. The histopathology of the boundary and status of the cervical lymph node metastasis were analyzed. Formation of abnormal hyperechoic halo and cervical lymph node metastasis in relation to age and gender were explored. The abnormal hyperechoic halo mainly represents cancer cell infiltration with reactive hyperplasia of inflammatory cells and fibrous tissue. In the hyperechoic halo group, the grayscale values for the nodular center, hyperechoic echo halo, and normal thyroid tissue were 1552.6 ± 578.6, 5792.0 ± 747.6, and 3582.7 ± 759.0, respectively (*P* < 0.05). The cervical lymph node metastasis rate was significantly lower in patients with hyperechoic halo (15.0%) than in those without (41.6%; *P* < 0.05) and significantly higher in those aged <45 years (53.3%) than in those aged ≥45 years (28.4%; *P* < 0.05). There were no significant correlations between gender and cervical lymph node metastasis or between age, gender, and hyperechoic halo formation (*P* > 0.05). cN0 PTMC patients with abnormal hyperechoic halo and age >45 years have a significantly reduced risk of cervical lymph node metastasis and relatively good prognosis.

## 1. Introduction

Papillary thyroid carcinoma (PTC) is the most common type of thyroid cancer, accounting for 70–90% of well-differentiated thyroid malignancies. Papillary thyroid microcarcinoma (PTMC) is a specific subgroup that is defined by the World Health Organization (WHO) as PTC with a maximum diameter of ≤1.0 cm. Most PTMCs are not detectable on clinical examination but diagnosed incidentally during pathologic examination after surgery. With the development of new sensitive devices and diagnostic procedures, the number of PTMC cases detected is increasing. A better understanding of its characteristics would facilitate better prevention and management of this disease [1, 2].

The boundary status of thyroid nodules has been one of the important ultrasound features in the diagnosis of thyroid cancer [3–5]. In recent years, with the continuous improvement of the resolution of ultrasonography, the detection rate of malignant thyroid nodules with abnormal hyperechoic halo has increased significantly. A large number of studies have reported the abnormal hyperechoic halo as one of the typical ultrasound signs in breast cancer, and the degree of malignancy of breast cancer with the above-mentioned sign is generally low [6]. However, reports on thyroid nodules with abnormally high echoes are relatively rare, and comprehensive and systematic studies of these nodules are lacking.

Lymph node metastasis has been one of the main predictors of recurrence in PTMC [7]. The recurrence risk in patients with lymph node metastasis can be 3.33 times that in patients without [8]. Previous studies suggested that according to the clinicopathological characteristics of PTMC, a correct evaluation of cervical lymph nodes is important to guide surgical treatment [9]. The basic consensus on the treatment options for patients with thyroid carcinoma lymph node metastases has been clearly defined by scholars and medical workers in both China and worldwide. However, there is still much controversy over the choice of treatment for patients with clinically cervical lymph node-negative (cN0) PTMC [10–12].

Therefore, the present study was carried out to investigate the pathological basis and prognostic value of abnormal hyperechoic halo in cN0 PTMC and to explore the correlations of age and gender with the formation of abnormal hyperechoic halo and cervical lymph node metastasis. The novelty of this study is it first describes the prognostic value of hyperechoic echo and risk of cervical lymph node metastasis in patients with cN0 PTMC. The results of this study provide useful guidance for the selection of appropriate treatment programs and provide a new idea for the preoperative evaluation of the risk of cervical lymph node metastasis in patients with cN0 PTMC.

## 2. Materials and Methods

### 2.1. Study Design and Settings

This was a single-center retrospective study. Clinical, imaging, and pathological data were collected and analyzed for patients who underwent ultrasonography for thyroid gland examination before surgical treatment for the first time in Zhoushan Hospital of Wenzhou Medical University and were confirmed to have single PTMC.

### 2.2. Clinical Data

Data for a total of 97 patients who fulfilled the inclusion criteria were collected from April 2016 to March 2017. Informed consent was obtained from all patients before undergoing ultrasonography, and the study was approved by the Ethics Committee of Zhoushan Hospital of Wenzhou Medical University.

The inclusion criteria included the following: (1) no suspicious swelling or structurally abnormal lymph nodes found before surgery and patients who underwent ultrasonography in our hospital within 2 weeks prior to surgery; (2) primary resection and central lymphadenectomy performed and single PTMC confirmed by pathology; and (3) no previous history of thyroid surgery, radiotherapy, or chemotherapy.

The exclusion criteria were as follows: (1) incomplete clinical, sonographic, or pathological data or poor ultrasound image quality; (2) inconsistency between the lesions found by ultrasound and pathology results; and (3) a pathological diagnosis of another type of thyroid cancer.

### 2.3. Equipment

Siemens Acuson S1000 and Acuson S2000 color Doppler ultrasound systems (Siemens AG, Munich, Germany) were used as the diagnostic apparatus, and a 9L4W linear array probe with transmitting frequency of 4-9 MHz was used. The mechanical index and gain were kept constant for all patients.

### 2.4. Scanning Method

The patient's neck was outstretched to fully expose the front of the neck. Thyroid nodules were scanned using conventional two-dimensional ultrasound to obtain the anatomical reference images. The nodule size, position, border, and lymph node metastasis were recorded.

### 2.5. Imaging Analysis

The ultrasound images of all patients were reviewed. Adobe Photoshop CS6 (PS) software (Adobe Systems Inc., San Jose, CA, USA) with a resolution of 300 dpi was used to acquire the grayscale values for the nodular center, hyperechoic echo halo, and normal thyroid tissue at the 3 and 9 o'clock positions of the cross and longitudinal section. Small squares were drawn, and the whole picture was equally divided. During measurement, we tried to maintain the top three regions at the same depth, and attention was paid to avoid large blood vessels, cystic degeneration, and calcification in the lesions. All sonographic examinations were performed by two sonographers (a chief physician and a master graduate student) independently. Both sonographers have received specialized scientific training and had relevant scientific research experience. Prior to the start of this study, the two sonographers underwent a consistency test with good consistency. When the grayscale values were inconsistent, two sonographers will remeasure together; otherwise, they will take the average values.

### 2.6. Pathological Analysis

A senior pathologist and an ultrasound master graduate student jointly reviewed the pathological sections of the included patients. The pathological components of the abnormal hyperechoic halo, the thickness and distribution of the halo, and cervical lymph node metastasis were observed and recorded.

### 2.7. Case Grouping

Based on the combined findings from imaging with pathological analysis, all cases were divided into two groups, the hyperechoic halo group and nonhyperechoic halo group.

### 2.8. Statistical Analysis

All statistical analyses were performed using SPSS version 19.0 software (SPSS Inc., Chicago, Illinois, USA). All quantitative data are reported as the mean ± standard deviation (SD). The differences in grayscale values among the nodular center, hyperechoic halo, and normal thyroid tissue were compared using one-way analysis of variance (ANOVA). Comparisons between groups were performed using the chi-square (*χ*^2^) test. *P* < 0.05 was considered statistically significant.

## 3. Results

### 3.1. Baseline Data

A total of 7 cases were excluded due to incomplete data. The main reason was that these patients had recently undergone ultrasound examinations in other hospitals and were subsequently treated in our hospital. To avoid wasting medical resources, no ultrasound was performed before surgery. No clear ultrasound data can be obtained. Ninety-seven patients were included, 81 were female and 16 were male, and their ages ranged from 26 to 75 years (49 ± 11 years). The tumor sizes ranged from 2 to 10 mm (5.9 ± 1.7 mm).

### 3.2. Measurement Results of Grayscale Values in Different Regions of Nodules in the Hyperechoic Halo Group

In the hyperechoic halo group, the grayscale values were 1552.6 ± 578.6, 5792.0 ± 747.6, and 3582.7 ± 759.0 for the nodular center, hyperechoic halo, and normal thyroid tissue, respectively, and the differences were statistically significant (*P* < 0.05; Table 1).

### 3.3. Pathological Components of the Abnormal Hyperechoic Halo

**Figure 1:**
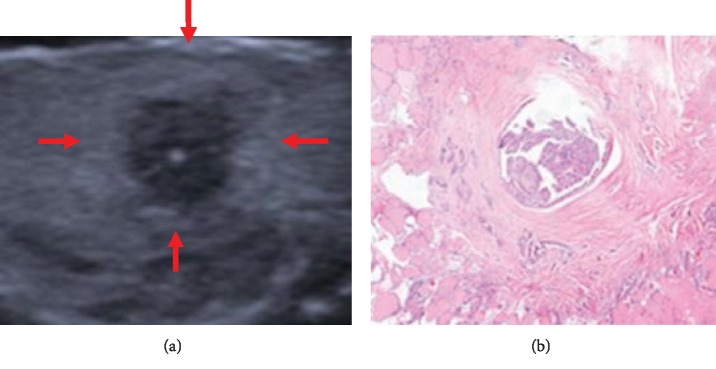
Hyperechoic halo nodule (a) observed by ultrasonography and (b) same nodule stained with hematoxylin and eosin (HE).

**Figure 2:**
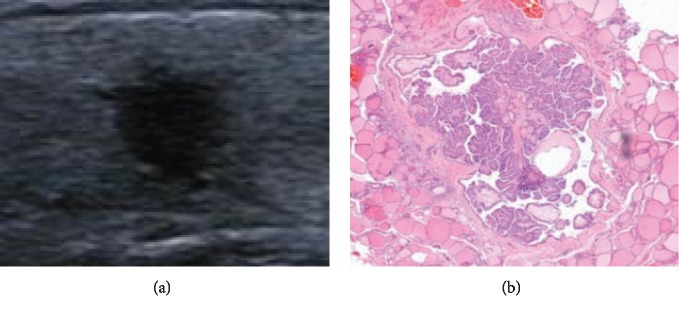
Nonhyperechoic halo nodule (a) observed by ultrasonography and the same nodule (b) stained with HE.

**Figure 3:**
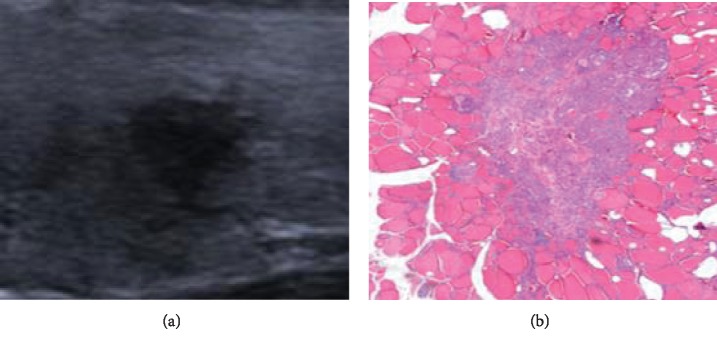
Nonhyperechoic halo nodule (a) observed by ultrasonography and same nodule (b) stained with HE.

Under the microscope, the main pathological components of the abnormal hyperechoic halo were cancer cell infiltration with reactive hyperplasia of inflammatory cells and fibrous tissue. Most of the malignant nodules were accompanied by the above-mentioned interstitial reactions, but their degrees varied. In this study, the pathological fiber ring of PTMC in the hyperechoic halo group was complete and thick (Figures 1(a) and 1(b)). The pathological fiber ring of PTMC in the nonhyperechoic halo group was partially complete but extremely thin (Figures 2(a) and 2(b)), and in the majority of cases, the internal fibrotic reaction within the lesion was significant but there was no obvious ring-like structure at the edge of the lesion or localized “fragmented” fibrosis around the lesion (Figures 3(a) and 3(b)). In other words, fibrosis around the nodules without hyperechoic halo often appeared as incomplete, uneven, thin, or absent.

### 3.4. Cervical Lymph Node Metastasis

Of the 97 patients with cN0 PTMC, 35 (36.1%) had cervical lymph node metastasis. The cervical lymph node metastasis rate in the hyperechoic halo group was significantly lower than that in the nonhyperechoic halo group (15.0% vs. 41.6%, respectively, *P* < 0.05; Table 2).

### 3.5. Correlation Analysis among Gender, Age, Abnormal Hyperechoic Halo Formation, and Cervical Lymph Node Metastasis

In addition to people under the age of 15, PTMC was observed in patients of all age groups. With increasing age, its incidence gradually increased until it peaked at 45–59 years and began to decline after 60 years (Table 3). There was no abnormal hyperechoic halo found in patients aged 15–29 years. The proportions of the above-mentioned ultrasound signs were 15.4%, 22.9%, and 26.3% in the 30–44-year-old, 45–59-year-old, and ≥60-year-old groups, respectively. The proportions of abnormal hyperechoic halo in males and females were 6.3% and 23.5%, respectively. However, there were no significant differences in the incidence rates of abnormal hyperechoic haloes among patients of different ages and genders (*P* > 0.05; Table 4), and there was also no significant difference in cervical lymph node metastasis between the genders (*P* > 0.05). However, the rate of cervical lymph node metastasis in those aged <45 years was significantly higher than that in those aged ≥45 years (*P* < 0.05; Table 5).

## 4. Discussion

Until recently, treatment for patients with PTMC remained controversial. With no consensus existing regarding its natural history, the recommended treatment has ranged from observation to total thyroidectomy with radioactive iodine ablation [13]. There have been an increasing number of patients undergoing total thyroidectomy, and while more aggressive therapy may be indicated for some cases, many may be overtreated [14]. Therefore, studies on relevant prognostic indicators are important for more accurate diagnosis and treatment planning.

Abnormal hyperechoic halo is a common ultrasound sign of malignant nodules. The abnormal hyperechoic halo at the boundary of breast nodules has been proven to be one of the typical ultrasonic signs of breast cancer [6]. In the present study, the above ultrasound signs also existed in some malignant thyroid nodules, while based on our clinical experience so far, abnormal hyperechoic halo has never been found on the border of benign thyroid nodules. This phenomenon may be related to the growth pattern of nodules: malignant nodules are dominated by invasive growth, whereas benign nodules are dominated by expansive growth.

Our results revealed that the pathological basis of abnormal hyperechoic halo of PTMC was cancer cell infiltration, which caused interstitial reactions at the edge of the lesion, and there was more reactive hyperplasia of inflammatory cells, such as neutrophils and lymphocytes, and fibrous tissue. Increased cell tissue composition can cause more scatter at the edges of the lesion and also can result in greater acoustic impedance differences between this area and the normal thyroid tissue. There is a relatively large amount of reflected energy in the edges of the lesion; thus, on a two-dimensional ultrasound image, the hyperechoic halo appears as a band-like abnormal hyperechoic region located between the simple cancer tissues and the pure thyroid tissue. Based on the combination of the ultrasound and pathology results, the pathological annulus fibrosus of PTMC in the hyperechoic halo group was complete and thick, whereas in the nonhyperechoic halo group, it often appeared as incomplete, uneven, thin, or absent. We believe that this phenomenon may be related to the degree of tumor cell activity and the patients' own condition. In the hyperechoic halo group, the tumor cells in each area have similar activity, and their invasiveness is relatively small. The tumor growth rate is relatively slow [15]. The body undergoes a sufficient interstitial reaction in the periphery of the lesion, wrapping the cancerous tissue within the nodule. In the nonhyperechoic halo group, the degree of malignancy of tumor cells is relatively high, and the tumor cells in different regions often have different levels of activity, while the extent of reactive hyperplasia of inflammatory cells and the amount of fibrous tissue are also not the same. No abnormal hyperechoic halo appeared on the ultrasound images and manifested as a complete thin fiber ring in the pathological images, which may be due to a weak interstitial response. That is, there was no significant increase in the extent of ultrasound scattering, or the difference in acoustic impedance between the increased pathological components and normal thyroid tissue was small. As a result, no abnormal hyperechoic haloes were detected in this area during the ultrasound examination. In this study, PS software was used to quantify the grayscale values of different areas of PTMC and further confirm the objective existence of abnormal hyperechoic haloes around PTMC. To minimize error, the sections containing the maximum diameter of the nodule were selected for grayscale value measurements and pathological analysis, and grayscale value measurements for the same nodule were performed at different regions.

Patients with lymph node metastasis have a high recurrence rate, and some cN0 PTMC patients are found to have lymph node metastasis after surgery [10]. In the present study, the cervical lymph node metastasis rate in the hyperechoic halo group was significantly lower than that in nonhyperechoic halo group. We propose bold assumptions that the abnormal hyperechoic halo is a protective factor against such lymph node metastasis in PTMC to some extent. This phenomenon may have multiple causes. On the one hand [16], in malignant nodules with hyperechoic halo, the tumor cells' reproductive ability is weak, and their growth rate is slower. On the other hand, hyperechoic haloes have a certain compression or restrictive effect on lymphatics around the tumor, limiting the transfer of cancer cells to the lymph nodes in the neck. We believe that malignant nodules with abnormal hyperechoic halo do not require immediate surgical treatment if the neck does not also show clearly metastatic lymph nodes. There are differences between our results and those of Zhu et al. [17]. The possible reason is that, although the abnormal hyperechoic halo is a manifestation of infiltration of cancer cells into the surrounding normal thyroid tissue, the present study mainly analyzed the relationship between abnormal hyperechoic haloes and cervical lymph node metastasis; that is, the focus of this study was different from that of Zhu et al.

PTMC shows a predilection for female patients. The results of this study also showed a nonlinear correlation between the incidence of PTMC and patient age. Among the 97 patients included, those aged 30–59 years had the highest occurrence of PTMC. This phenomenon may be related to the pressure of work and life of people in this age group in modern society, and factors such as diet and irregular work habits of these people, or it may also be related to hormone levels in young people. This is similar to the results of the studies by Zhou et al. and Li et al. [18, 19]. However, the cervical lymph node metastasis rate gradually decreased as the patients' age increased, and this was similar to the results of studies by Ito et al., Li et al., and Xu et al. [7, 20, 21]. According to the age of patients, all selected cases were further divided into two groups, the <45-year-old group and the ≥45-year-old group. The results showed that the rate of cervical lymph node metastasis was significantly higher in the <45-year-old group than in the ≥45-year old group. This is consistent with the results of previous studies conducted by Lu et al. and Vriens et al. [22, 23]. The reasons for the above results are not yet clear and may be related to the faster metabolism of young patients. In addition, in this study, the cervical lymph node metastasis rate in male patients was slightly higher than that in females (43.8% for males vs. 34.6% for females), but the difference was not statistically significant. This is consistent with a report by Wu et al. [24]. The incidence of PTMC is higher in female patients than in male patients, while males are considered to have a higher risk for lymph node metastasis [7, 25]. The possible reason for this may be that females have higher levels of estrogen and progesterone, which affect the levels of pituitary gonadotropin-releasing hormone, whereas the higher basal metabolic rate in males may incite an overactive proliferation of tumor cells [7, 26, 27]. The incidence in males may also be associated with their living environment, in which progression to malignancy and lymph node metastasis may occur once the protection mechanism is damaged [22]. Recent studies suggested that male gender is an independent prognostic factor for recurrence in PTC > 1 cm, but not in PTMC [28].

Our results revealed that hyperechoic haloes begin to be found in patients over 30 years old. The incidence rates of hyperechoic haloes in the 30–44-year-old group, the 45–59-year-old group, and the ≥60-year-old group gradually increased, but there were no significant differences among these three groups. The rate of hyperechoic halo in males was lower than that that in females, but there was no significant correlation between gender and the formation of hyperechoic haloes. In other words, an abnormal hyperechoic halo may more likely to be found in the nodule boundary in female and older patients, but the correlation is not statistically significant.

In conclusion, the abnormal hyperechoic halo is a manifestation of cancer cell infiltration into the surrounding normal thyroid tissue. Nodules with abnormal hyperechoic halo generally indicate a high likelihood of malignancy, but this kind of nodule has a relatively small risk of cervical lymph node metastasis and a relatively good prognosis. Therefore, in clinical practice, follow-up observation of patients with the above-mentioned ultrasound signs is needed in order to improve the quality of life of patients. In addition, patient age may also have some predictive value for cervical lymph node metastasis in these patients. However, although the proportions of hyperechoic halo at different ages and genders were not the same, there was no significant correlation between the occurrence of abnormal hyperechoic halo and the age and gender of patients.

Our study had some limitations. The number of cases with hyperechoic halo was significantly lower than that without. Moreover, all selected cases had malignant nodules with PTMC, and the pathological types were relatively single. In addition, there were few male patients in the whole group, and so far, the abnormal hyperechoic haloes shown on the sonogram have not yet formed mature quantitative indicators. Nonetheless, the results of this study warrant further exploration with a larger sample size.

## Figures and Tables

**Table 1:** Comparison of grayscale values in different regions of nodules in the hyperechoic group.

Position	Number of cases	Grayscale value
Nodule center	20	1552.6 ± 578.6^#^
Hyperechoic halo	20	5792.0 ± 747.6^∗^^#^
Normal thyroid tissue	20	3582.7 ± 759.0^∗^

Note: data are presented as the mean ± standard deviation. ^∗^*P* < 0.05 compared with the nodular center; ^#^*P* < 0.05 compared with normal thyroid tissue.

**Table 2:** Comparison of cervical lymph node metastasis in hyperechoic and non-hyperechoic halo groups (number of cases).

Group	Cervical lymph node metastasis		*χ* ^2^	*P*
	Yes	No		
Hyperechoic halo group (*n* = 20)	3 (15.0%)	17 (85.0%)	4.856	0.028
Nonhyperechoic halo group (*n* = 77)	32 (41.6%)	45 (58.4%)		

Note: data are presented as the mean ± standard deviation or number of patients (*n*) with percentage (%).

**Table 3:** The percentage of PTMC in all age groups.

Age (years)	PTMC (*n* = 99)	Percentage (%)
<15	0	0%
15-29	4	4.0%
30-44	26	26.3%
45-59	48	48.5%
≥60	19	19.2%

Note: data are presented as the number of patients (*n*) with percentage (%).

**Table 4:** The formation of hyperechoic halo in PTMC patients of different ages and genders (number of cases).

Group		PTMC (*n* = 99)		*χ* ^2^	*P*
		Hyperechoic halo group (*n* = 20)	Nonhyperechoic halo group (*n* = 77)		
Age, years	30-44	4 (15.4%)	22 (84.6%)	1.504	0.471
	45-59	11 (22.9%)	37 (77.1%)		
	≥60	5 (26.3%)	14 (73.7%)		

Gender	Male	1 (6.3%)	15 (93.7%)	1.480	0.224
	Female	19 (23.5%)	62 (76.5%)		

Note: data are presented as the number of patients (*n*) with percentage (%).

**Table 5:** Comparison of cervical lymph node metastasis in PTMC patients of different ages and genders (number of cases).

Group		PTMC (*n* = 99)		*χ* ^2^	*P*
		Cervical lymph node metastasis (*n* = 35)	No cervical lymph node metastasis (*n* = 62)		
Age (years)	<45	16 (53.3%)	14 (46.7%)	5.604	0.018
	≥45	19 (28.4%)	48 (71.6%)		

Gender	Male	7 (43.8%)	9 (56.2%)	0.488	0.485
	Female	28 (34.6%)	53 (65.4%)		

Note: data are presented as the number of patients (*n*) with percentage (%).

## Data Availability

No additional unpublished data are available.
